# A Plasmonic Optoelectronic Resistive Random‐Access Memory for In‐Sensor Color Image Cryptography

**DOI:** 10.1002/advs.202403043

**Published:** 2024-05-29

**Authors:** Quan Yang, Yu Kang, Cheng Zhang, Haohan Chen, Tianjiao Zhang, Zheng Bian, Xiangwei Su, Wei Xu, Jiabao Sun, Pan Wang, Yang Xu, Bin Yu, Yuda Zhao

**Affiliations:** ^1^ College of Integrated Circuits Hangzhou Global Scientific and Technological Innovation Centre Zhejiang University 38 Zheda Road Hangzhou 310027 China; ^2^ Research Center for Frontier Fundamental Studies Zhejiang Lab Hangzhou 311100 China; ^3^ Micro‐Nano Fabrication Center Zhejiang University 38 Zheda Road Hangzhou 310027 China; ^4^ College of Optical Science and Engineering Zhejiang University Hangzhou 310027 China; ^5^ Key Laboratory of Optoelectronic Chemical Materials and Devices of Ministry of Education Jianghan University Wuhan 430056 China

**Keywords:** 2D materials, cryptography, in‐sensor computing, optoelectronic RRAM, plasmonic

## Abstract

The optoelectronic resistive random‐access memory (RRAM) with the integrated function of perception, storage and intrinsic randomness displays promising applications in the hardware level in‐sensor image cryptography. In this work, 2D hexagonal boron nitride based optoelectronic RRAM is fabricated with semitransparent noble metal (Ag or Au) as top electrodes, which can simultaneous capture color image and generate physically unclonable function (PUF) key for in‐sensor color image cryptography. Surface plasmons of noble metals enable the strong light absorption to realize an efficient modulation of filament growth at nanoscale. Resistive switching curves show that the optical stimuli can impede the filament aggregation and promote the filament annihilation, which originates from photothermal effects and photogenerated hot electrons in localized surface plasmon resonance of noble metals. By selecting noble metals, the optoelectronic RRAM array can respond to distinct wavelengths and mimic the biological dichromatic cone cells to perform the color perception. Due to the intrinsic and high‐quality randomness, the optoelectronic RRAM can produce a PUF key in every exposure cycle, which can be applied in the reconfigurable cryptography. The findings demonstrate an effective strategy to build optoelectronic RRAM for in‐sensor color image cryptography applications.

## Graphical abstract

The optoelectronic Au/h‐BN/Au and Ag/h‐BN/Au resistive random‐access memory (RRAM) array is developed to achieve high‐quality in‐sensor color image cryptography. Resistance switching characteristics of RRAM devices can be modulated by optical stimuli due to plasmonic effects of noble metals. The double‐binary physically unclonalbe function keys can be simultaneously generated in the optoelectronic RRAM array via reading the high resistance state.

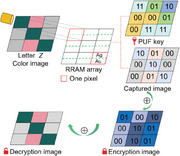

## 1 Introduction

In‐sensor computing is an emerging field that aims to bring data storage and processing capabilities to the sensors.^[^ ^1^ ^]^ The preprocessing of image data in the photodetectors have been demonstrated to successfully minimize the data transfer and speed up the data processing in an energy‐efficient way.^[^ ^2^ ^]^ Another important application of in‐sensor computing is the integration of cryptography in the image sensors to guarantee the image information security.^[^ ^3^ ^]^ Different from algorithm‐based image cryptography, hardware‐level information security plays an important role against the attack from large‐scale machine learning. And the conventional hardware‐based image cryptography is composed of separated sensing and encryption units, which suffers from threats during the data transmission. In‐sensor image cryptography can simultaneously realize the photodetection and physically unclonable function (PUF) in a single sensor device.^[^ ^4^ ^]^ The widely adopted device structures include optoelectronic transistor and optoelectronic resistive random‐access memory (RRAM). Optoelectronic transistors normally generate a static PUF key, which is coupled with the image information.^[^ ^3^, ^5^ ^]^ Optoelectronic RRAM has the intrinsic and high‐quality randomness in resistance and voltage distribution, which is the desirable hardware to realize the dynamic reconfigurable PUF.^[^ ^6^ ^]^ To achieve the in‐sensor cryptography, it is highly promising to fabricate optoelectronic RRAM, in which the generated PUF keys are reconfigurable and decoupled with image information.

The mechanism of optoelectronic RRAMs lies in the interaction between photo‐induced carriers in the resistive switching layer (e.g., metal oxide,^[^ ^2^, ^7^ ^]^ 2D materials^[^ ^8^ ^]^ and perovskite^[^ ^9^ ^]^) and the filament growth dynamics.^[^ ^8b^ ^]^ However, the light‐matter interaction is relatively weak and the photo‐generated carriers have to migrate through the resistive switching layer to interact with the conductive filament. Noble metals, especially gold (Au) and silver (Ag) display strong light–matter interaction and exhibit tunable optical properties due to their surface plasmon resonance (SPR). In optoelectronic RRAM, SPR‐generated hot carriers and the photothermal effect can efficiently affect the photochemical and photoelectric dynamic of conductive filament, resulting in the optical‐controlled resistive switching behavior. Especially for Cu,^[^ ^10^ ^]^ Ag,^[^ ^11^ ^]^ and Al^[^ ^12^ ^]^ noble metals in conductive bridge RRAM, the light interaction and metal filament growth are strongly coupled together.^[^ ^13^ ^]^ The hot electrons and the photothermal effect generated from active metal electrode will have direct interaction with conductive filament without its migration. Furthermore, by adopting different noble metals, plasmonic optoelectronic RRAM devices display wavelength tunability and establish the foundation for color recognition, which is relatively unexplored. And the color image cryptography is an important application because the color information can provide rich discriminative clues for visual inference. Thereby it would be interesting to introduce SPR into RRAM, enabling the development of functional optoelectronic RRAM with nonvolatile color image sensing, storage ability and reconfigurable PUF generation.^[^ ^3^, ^14^ ^]^

In this paper, the optoelectronic RRAM arrays with semitransparent noble metal as electrodes have been fabricated to perform the in‐sensor color image cryptography. Two kinds of noble metals Ag and Au are selected as the top electrodes to respond to blue and red light, respectively. The resistive switching (RS) layer is the few‐layer hexagonal boron nitride (h‐BN) and the inert Au film works as the bottom electrode. The color images were captured by comparing low resistance state (LRS) currents during SET process under dark and illumination conditions. And the reconfigurable PUF keys were harvested in every exposure cycle through reading the high resistance state (HRS) resistance due to their high random distribution. Due to the different absorption peak of Ag and Au semitransparent electrodes, RRAM devices can be utilized to mimic the biological dichromatic cone cells to perform the color perception. The photoresponse ability originates from the plasmonic effect of top noble metals, which facilitates the rupture of conductive filament. Thus, our optoelectronic RRAM arrays can generate reconfigurable PUF keys and capture color images in every exposure cycle, leading to the high‐quality in‐sensor color image cryptography.

## 2 Results and Discussion

### 2.1 Optoelectronic RRAM for Color Image Capture

Dichromatism in mammals displays good performance in the accurate pattern recognition and the color can be recognized by mixing two identified wavelengths. Inspired by the dichromatism, optoelectronic devices have been developed to realize the dual‐wavelength color image perception. Due to the wavelength selectivity of different noble metals, it is possible to change the top metal material to modulate the response wavelength of optoelectronic RRAM for in‐sensor color image cryptography. We choose the noble metal Ag and Au as the semitransparent top electrode and the response wavelength is ≈400 and ≈600 nm, respectively. Combined Au/h‐BN/Au RRAM with Ag/h‐BN/Au RRAM, the RRAM device arrays can capture the binary color image and simultaneously realize the corresponding encryption.

**Figure 1:**
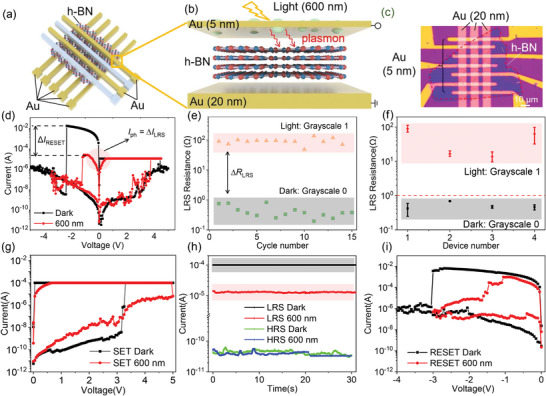
Schematic of a) the Au/h‐BN/Au optoelectronic RRAM device array and b) the device structure. c) Photograph of the optoelectronic RRAM array. d) *I*–*V* curves of the RRAM modulated by 600 nm light. e) LRS currents of the RRAM under light and dark conditions. f) Device‐to‐device distribution of LRS. g) SET under dark and light. h) Read of LRS current and HRS current after different light irradiation conditions. i) RESET under dark and light.

**Figure** **1**a–c exhibits the schematic structure and the photograph of the 3 × 5 h‐BN based optoelectronic RRAM array, composed of a few‐layer h‐BN sheet sandwiched between the top semitransparent Au electrode and the bottom inert Au electrode (device size 5 × 10 µm^2^). The thickness of the h‐BN layer, top Au electrode, and bottom Au electrode is ≈7, 5, and 20 nm, respectively. The thickness of the top Au electrode has been optimized to exhibit the plasmonic effect while maintaining the resistance switching behavior. We carry out the optoelectronic RS test by grounding the bottom Au electrode and applying the voltage to the top Au electrode and the light wavelength is 600 nm with the light intensity of 55 mW cm^−2^. The current‐voltage (*I*–*V*) curves collected under dark and light conditions are presented in Figure 1d and Figure S1 (Supporting Information). Under the dark condition, a compliance current *I*_cc_ of 10^−5^ A has been SET during the positive voltage sweep, which can prevent the permanent breakdown of the h‐BN layer. The *I*–*V* curves show a stable asymmetric bipolar RS behavior with a relatively high RESET current *I*_RESET_ of more than 10^−2^ A. Compared with *I*_cc_ of 10^−5^ A, the high *I*_RESET_ indicates that the rupture of the robust conductive filament is quite difficult when solely replying on the voltage‐driven ion migration. The excessive Joule heating is needed to induce the local rupture of the conductive filament during the RESET process. Under light irradiation, the *I*–*V* curve displays a symmetric feature with a dramatically reduced *I*_RESET_ to 10^−5^ A. The extremely large difference of the RESET current Δ*I*_RESET_ in the RESET process indicates that light stimulus can effectively affect the evolution dynamics of the filament growth. Therefore, LRS resistance can be used to record the image information. Another important feature of our optoelectronic RRAM lies in the HRS resistance independent on the external light stimulation. The significantly intrinsic random distribution of HRS resistance varies from 10^4^ to 10^8^ Ω, which can be used to generate the reconfigurable PUF keys.

Figure 1e shows the cycle‐to‐cycle stability of the optoelectronic RRAM, which is extracted from the 100 direct current *I*–*V* cycles (Figure S2a, Supporting Information). The LRS resistance of the optoelectronic RRAM was ≈0.1 and ≈100 Ω under dark and light irradiation, respectively. Figure 1f illustrates the stable device‐to‐device distribution of LRS resistance under dark and light irradiation, respectively. The LRS resistance under dark varies from 0.1 to 1 Ω, and the LRS resistance under light varies from 10 to 100 Ω. These devices do not exhibit significant variation, demonstrating excellent cycling stability and minimal device‐to‐device variation.

To study the light modulation effect on RS behaviors, the optical stimulus has been irradiated on the devices during SET or RESET process separately. When light irradiation is only applied during the SET process, the LRS current is smaller than that under dark (Figure 1g). To exclude the contribution of photocurrent, the low resistance state can be read after setting to LRS and it still displays a significant difference from the control sample (Figure 1h). And the LRS resistance shows the good retention for over 30 s. When light irradiation is only applied during the RESET process (Figure 1i), the LRS resistance is almost the same. It is obvious that the RESET voltage is small under light and the HRS resistance is comparable with that under dark. It demonstrates that light irradiation can impede the filament growth during the SET process and promote the filament dissolution during the RESET process. Other test conditions, e.g., SET under dark and RESET under light, and SET under light and RESET under dark were also performed as shown in Figures S3 and S4 (Supporting Information), which shows the light‐modulation on LRS current for image capture.

**Figure 2:**
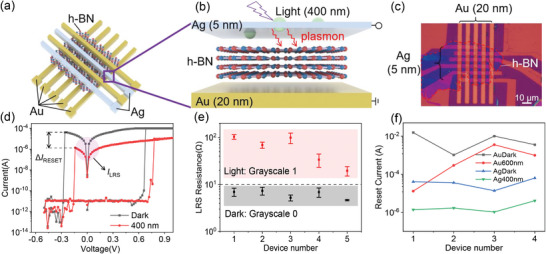
Schematic of Ag/h‐BN/Au optoelectronic RRAM a) array and b) single device. c) Photograph of the optoelectronic RRAM array. d) *I‐*‐*V* curves of the RRAM modulated by light with wavelength of 400 nm. e) Device‐to‐device distribution of LRS with/without light irradiation. f) RESET current of Ag/h‐BN/Au and Au/h‐BN/Au under dark and light, respectively.

**Figure** **2**a shows the schematic structure of the Ag/h‐BN/Au RRAM by using 5‐nm‐thick Ag as the top electrode. The device size is 5 × 5 µm^2^ and the h‐BN thickness is ≈7 nm. We carried out the optoelectronic RS test by grounding the bottom Au electrode and applying the voltage to the top Ag electrode. The light wavelength is 400 nm with the light intensity of ≈53 mW cm^−2^. Figure 2c and Figure S5 (Supporting Information) display the *I*–*V* curves under dark conditions and upon exposure to light. Compared with Au/BN/Au optoelectronic RRAM, the Ag/BN/Au optoelectronic RRAM shows the symmetric bipolar nonvolatile RS curves. The RESET currents (*I*_RESET_) under both dark and light conditions are lower than *I*_cc_. This is because the electric field is enough to fracture the active metal Ag conductive filament during the RESET process. Based on the LRS current *I*_LRS_, Δ*I*_RESET_ and RESET voltage in *I*–*V* curves, we can conclude that the optical stimulus introduces the similar function in Ag/BN/Au optoelectronic RRAM, which is the impedance of filament growth and the promotion of filament annihilation. In addition, the HRS current shows no obvious change under the light irradiation and has the digital RS characteristic as shown in Figure S6a (Supporting Information).

Figure 2e illustrates the stable device‐to‐device distribution of LRS resistance under dark and light irradiation. The LRS resistance under dark varies from 1 to 10 Ω, and the LRS resistance under light varies from 10 to 100 Ω. Figure 2f illustrates the stable device‐to‐device distribution of RESET current under dark and light irradiation of Ag/BN/Au and Au/BN/Au RRAM. The RESET current of Ag/BN/Au RRAM is smaller than that of Au/BN/Au RRAM. These devices do not exhibit significant variation, demonstrating excellent cycling stability and minimal device‐to‐device variation.

**Figure 3:**
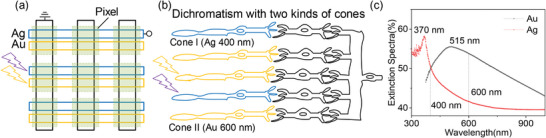
a) Optoelectronic Ag/h‐BN/Au and Au/h‐BN/Au RRAM array. b) Two type of cone cells in the dichromatism. c) The extinction spectra of Ag/h‐BN/Si and Au/h‐BN/Si structure.

Our plasmonic optoelectronic Ag/h‐BN/Au and Au/h‐BN/Au RRAM array can mimic two types of cone cells in dichromatism mammalian for the color perception by responding to their characteristic wavelength (Figure 3a,b). Figure 3c shows the extinction spectra of Ag/h‐BN/Si and Au/h‐BN/Si structures with a pronounced absorption peak at 370 and 515 nm, respectively. The light absorption at the wavelength of 400 nm (600 nm) is higher than 50% for the Ag/h‐BN/Si (Au/h‐BN/Si) structure, which can carry the color information being purple (yellow). The two wavelengths of 400 and 600 nm are close to the absorption peak of cone cells in the human vision system (420 and 558 nm). Therefore, light with different wavelengths can induce significant differences in LRS conductance (Figures 1f and 2e) in Ag/h‐BN/Au and Au/h‐BN/Au RRAM devices, indicating the capability of color selectivity of our optoelectronic RRAM array. Specifically, two stand‐alone Ag/h‐BN/Au and Au/h‐BN/Au devices are integrated to form one pixel, which can respond to two light wavelengths of 400 and 600 nm. Furthermore, the obvious differentiation in the RESET current (Figure 2f) enables the color perception by the optoelectronic RRAM array. In conclusion, the optoelectronic Ag/h‐BN/Au and Au/h‐BN/Au RRAM arrays have color selectivity, which is similar to the two types of the biological cone in dichromatism. And it is possible to mimic trichromatic and tetrachromatic color visions by tuning the plasmonic characteristic wavelength of the top metal electrode.

### 2.2 Switching Mechanism of Plasmonic Optoelectronic RRAM

**Figure 4:**
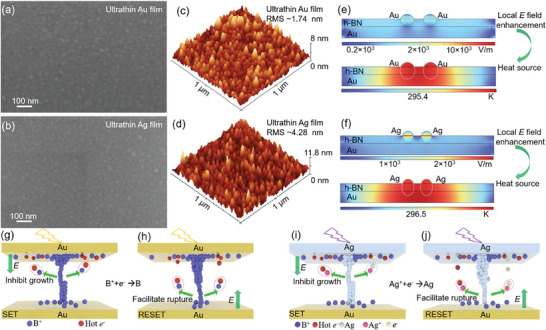
SEM images of top electrode a) Au and b) Ag film. AFM images of top electrode c) Au and d) Ag film. Thermal distribution and corresponding electric field distribution of e) Au/BN/Au and f) Ag/BN/Au RRAM. Optoelectronic RS during g) SET and h) RESET of Au/BN/Au RRAM; and during i) SET and j) RESET of Ag/BN/Au RRAM.

The surface morphologies of Au and Ag ultrathin film have been characterized by scanning electron microscopy (SEM) and atomic force microscopy (AFM). **Figure** **4**a,b presents the SEM image of 5 nm‐thick Au and Ag thin film utilized as the semitransparent top electrode in optoelectronic RRAMs, respectively, in which the obvious nanoparticles are observed. Through the statistical analysis of the nanoparticle size, the mean diameter of nanoparticle is ≈18 and ≈14 nm for Au and Ag film, respectively. The mean spacing between the adjacent nanoparticles is also measured according to SEM images in Figure S7 (Supporting Information). In our work, the small‐sized nanoparticles are obtained mainly due to the high roughness of extremely thin metal film through magnetron sputtering deposition. Figure 4c,d shows the 3D AFM images of Au and Ag thin films, and Figures S8 and S9 (Supporting Information) show their surface root mean square (RMS) roughness of ≈1.74 and ≈4.28 nm in a 1 µm^2^ area, respectively. The rough surface exists the noncontinuous grain that has contribution to the localized surface plasmon resonances.^[^ ^15^ ^]^ In contrast, the AFM images of the bottom Au/Ti electrode in Figure S10 (Supporting Information) display an ultra‐smooth surface with the RMS roughness of ≈0.677 nm.

Based on the device structure and the surface morphology of top electrodes and bottom electrodes, we performed numerical simulations via the finite element method to investigate the plasmonic effect in our RRAM device. The model describes as the plasmonic nanoparticle‐on‐mirror nanocavities in which the nanoscale gap between the nanoparticle and ultra‐smooth Au film is formed by few‐layer h‐BN (≈7 nm thick, Figure S11, Supporting Information). And the top Au or Ag ultrathin film with corrugated surface can be regarded as a chain of semi‐cylinder nanoparticles and the Au/Ti bottom electrode can be considered as the ultra‐smooth mirror. The local electric field (Figure 4e,f) is distributed in the Au substrate (bottom electrode) and at the bottom of Ag or Au nanoparticles (top electrode).^[^ ^16^ ^]^ The local electric field enhancement can cause the photothermal effect (Figure 4e,f) and the isolated Ag and Au nanoparticles will be the major heat source to increase the temperature.^[^ ^15^, ^17^ ^]^ The increased temperature near Ag and Au nanoparticles has the significant contribution to the rupture of conductive filament, since the position of the conductive filament is close to the top metal electrodes. By comparing the *I‐*‐*V* curves under dark and light conditions (Figures 1d and 2d), we can exclude the Joule heating effect on the filament dynamics under light irradiation due to the greatly reduced reset current. Thus, our plasmonic optoelectronic Au/BN/Au and Ag/BN/Au RRAM display extreme light–matter interactions due to the local electric field enhancement and the corresponding photothermal effect, leading to the light‐promoted rupture of conductive filament.

In addition to the photothermal effect, the photogenerated hot electrons also contribute to the rupture of conductive filament in our plasmonic optoelectronic RRAM. In Au/BN/Au plasmonic RRAM, the conductive filament is boron vacancies.^[^ ^18^ ^]^ During the SET process under light illumination (Figure 4g), the photogenerated hot electrons accumulate near the top Au electrode under the drive of external electric field. These photogenerated hot electrons interact with the boron ions, which inhibit the growth of boron vacancy conductive filament. Thus, light stimulation can increase the LRS resistance. During the RESET process (Figure 4h), under the reverse electric field driving, the photogenerated hot electrons can facilitate the rupture of conductive filament. In Ag/BN/Au optoelectronic RRAM, the Ag conductive filament have the vital contribution to the RS behaviors. And the photogenerated hot electrons (Figure 4i) inhibit the oxidation of Ag atom. During the RESET process (Figure 4j), the photogenerated hot electrons can facilitate the oxidation of Ag atom.

### 2.3 In‐Sensor Color Image Encryption and Decryption

**Figure 5:**
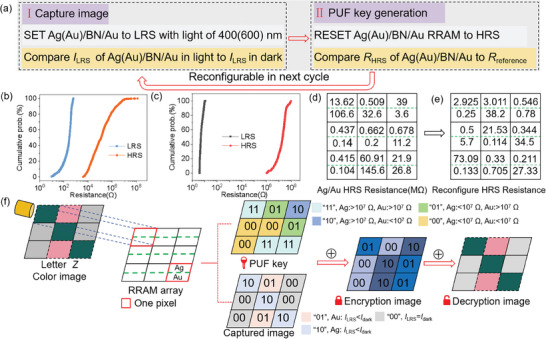
a) Flowchart of in‐sensor color image cryptography in optoelectronic RRAM arrays. Resistance distribution in HRS of b) Au/BN/Au and c) Ag/BN/Au RRAM. d,e) Harvest HRS resistance for reconfigure PUF. f) Image capture and PUF generation for encryption and decryption.

The optoelectronic RRAM array can capture color images due to the surface plasmon resonance effect of noble metal (Ag and Au) with its wavelength tunability. Two stand‐alone Ag/h‐BN/Au and Au/h‐BN/Au devices are integrated to form one pixel, which can respond to two light wavelengths of 400 and 600 nm. This pixel structure is similar to CMOS image sensor, where each pixel consists of three subpixels (red, green and blue). And the intrinsic and high‐quality randomness in RRAM arrays produce the reconfigurable PUF keys for image cryptography. Thus, our optoelectronic RRAM array can perform the in‐sensor color image cryptography, in which the produced key is used to encrypt the captured color image. The operation method of our device array can be described as follows (**Figure** **5**a): 1) SET the RRAM to LRS under light of 400 and 600 nm. Read the photocurrent to capture the color image. 2) RESET the device to HRS. Read the HRS resistance to generate the reconfigurable PUF key.

In previous works about RRAM based PUF,^[^ ^3^, ^6^, ^19^ ^]^ various parameters (e.g., RESET voltage, LRS resistance) are obtained as the PUF keys. In this work, the HRS resistance is harvested for generating PUF because the HRS resistance varies more significantly and the PUF generation process decouples with image capture process. As shown in Figure 5b,c, the HRS resistance values vary from 10^4^ to 10^8^ Ω in Au/h‐BN/Au RRAM, and range from 10^6^ to 10^8^ Ω in Ag/h‐BN/Au RRAM. The double‐binary PUF key was generated through comparing readout HRS resistance of both devices in one pixel with the reference resistance of 10 MΩ. Figure 5d shows the obtained HRS resistance, which can be reconfigured in next cycle (Figure 5e). If the resistances of both devices are larger than the reference resistance, it is donated as bits “11”. When the resistances of both devices are smaller than the reference resistance, it is donated as bits “00”. If only one RRAM device displays an HRS resistance larger than 10 MΩ, it is regarded as bits “01” (*R*_HRS_ of Au/h‐BN/Au > 10 MΩ) or “10” (*R*_HRS_ of Ag/h‐BN/Au > 10 MΩ). To quantitatively evaluate the PUF key performance, randomness and irreproducibility have been characterized using uniformity, uniqueness and stability (Supplementary Notes). The Hamming weight of our device indicates the uniformity of the PUF key with the value of 0.5, which is the ideal value of the Hamming weight of 0.5. The uniqueness and stability are 0.5 and 0.903, respectively, which is close to the ideal value of 0.5 and 1 (Figures S12 and S13, Supporting Information).

Figure 5f schematically illustrates the color image capture, image encryption and decryption. A 3 × 3 optoelectronic RRAM array was fabricated and each pixel contained two RRAM devices using the Ag and Au as the top electrode, respectively. The RRAM device array has been exposed to the image of letter *Z* with a binary color of ≈400 and ≈600 nm (Figure 5f). The LRS current (*I*_LRS_) is compared with dark current, obtaining the photocurrent and the color. The captured color image can be further converted into a double‐binary bit array. If both devices in one pixel display the negligible photocurrent, it is donated as bits “00”. When both devices in one pixel show the obvious photocurrent, it is donated as bits “11”. If only one device in the pixel has the photocurrent, it is donated as bits “01” (photoresponse from Au/h‐BN/Au) or “10” (photoresponse from Ag/h‐BN/Au). Then, the device has been RESET to HRS and the HRS resistance is used as the PUF key. The Vernam algorithm in symmetric cryptography is used to encrypt and decrypt images. The exclusive OR operation between captured image and PUF key was performed as shown in Figure 5f. The PUF keys have a good performance to perform the symmetric cryptography. Therefore, the device array can simultaneously capture color image and generate PUF key. It should be noted that an experimental XOR operation scheme^[^ ^6^, ^20^ ^]^ is proposed through the circuit‐level parallel connection of two optoelectronic RRAM with a series connection load resistance as shown in Figure S14 (Supporting Information). Before the transmission over the public network, the sender encrypts the image with our security PUF key to generate the cipher. An authorized receiver can decrypt the cipher (secured information) with the same PUF key. Thus, PUF based in‐sensor color image cryptography, operated under the light and electric stimuli, can achieve the balanced performance between high security protocol and high efficiency. Our h‐BN based RRAM array was fabricated as the prototype to demonstrate the in‐sensor color image cryptography. In the future work, the large‐area h‐BN grown by chemical vapor deposition can be adopted to fabricate wafer‐scale RRAM crossbar arrays. The detailed processes including h‐BN transfer, etching, lithography and metal deposition are CMOS‐compatible, displaying high scalability.^[^ ^21^ ^]^

## 3 Conclusion

We fabricated the h‐BN based plasmonic optoelectronic RRAM with the noble metal Ag and Au as the top electrode to perform the in‐sensor color image cryptography. The light irradiation can enhance the annihilation of conductive filaments and induce the optically controlled RS switching, which was used to capture image and color recognition. The photo sensing is based on the plasmonic effect of top electrode noble metal Ag and Au with characteristic wavelengths of 400 and 600 nm, respectively. The reconfigurable double‐binary PUF keys were harvested through comparing the HRS resistance with reference resistance due to the randomness variation. The in‐sensor color image cryptography has been demonstrated by converting the image intensity and color into a double‐binary bit array. The unique device structure and the pixel design in our work provided a feasible solution for the encryption and decryption of both image intensity and color information, which lays foundation for multi‐mode information cryptography. And in‐sensor cryptography can bridge the sensory terminal with the security hardware and enhance the security protocols.

## 4 Experimental Section

### Materials

Bulk carbon‐doped h‐BN single crystals were grown at atmospheric pressure and high temperature using a metal alloy solvent. The h‐BN powder was mixed with the Ni–Cr alloy in a cylindrical crucible. The carbon powder was added to tune the vacancy concentration. The weight ratio of the alloy, h‐BN powder and carbon powder is 100:10:0.6. In Au/h‐BN/Au RRAM, the vacancies dominant the formation of conductive filament. And in Ag/h‐BN/Au RRAM, the vacancies in h‐BN can promote the Ag ion migration, leading to a low forming voltage and the improved device performance.

### Device Fabrication and Characterization

The h‐BN flakes were mechanically exfoliated by the scotch‐tape method, transferred to PVA, and then transferred onto the Au bottom electrode. The thickness of h‐BN flakes was monitored by an optical microscope and confirmed by AFM. Au(Ag)/h‐BN/Au RRAM were fabricated by direct‐write lithography, Au(20 nm)/Ti(5 nm) bottom electrode on the Si/SiO_2_ substrate, and Au(5 nm) and Ag(5 nm)/ITO(15 nm) top electrodes, by sputtering evaporation at a base pressure of ≈5 × 10^−6^ Torr and a deposition rate of 1 nm s^−1^ for Au and a deposition rate of 0.35 nm s^−1^ for Ag. The lift‐off process was carried out in acetone. All the devices were electrically characterized in an integrated optoelectronic test platform including probe station, continuous adjustable laser source, optical power meter, and Keithley 2636.

## Conflict of Interest

The authors declare no conflict of interest.

## Supporting information

Supporting Information

## Data Availability

The data that support the findings of this study are available from the corresponding author upon reasonable request.
