# Supplementary material for: A Plasmonic Optoelectronic Resistive Random‐Access Memory for In‐Sensor Color Image Cryptography
Source: Adv Sci (Weinh). 2024 May 29;11(29):2403043. doi: 10.1002/advs.202403043 (PMC11304321; doi:10.1002/advs.202403043)
Supplement: Supplementary file 1 — Supporting Information [file ADVS-11-2403043-s001.docx]

Supporting Information

A Plasmonic Optoelectronic RRAM for In-Sensor Images Cryptography

Quan Yang,^1^ Yu Kang, ^1^ Cheng Zhang, ^1^ Haohan Chen, ^1^ Tianjiao Zhang, ^1^ Zheng Bian, ^1^ Xiangwei Su,^1^ Wei Xu, ^2^ Jiabao Sun,^3^ Pan Wang, ^4^ Yang Xu, ^1^ Bin Yu, ^1^ and Yuda Zhao^*1,5^

^1^ College of Integrated Circuits, Hangzhou Global Scientific and Technological Innovation Centre, Zhejiang University, 38 Zheda Road, Hangzhou 310027, China.

^2^ Research Center for Frontier Fundamental Studies, Zhejiang Lab, Hangzhou, China

^3^ Micro-Nano Fabrication Center, Zhejiang University, 38 Zheda Road, Hangzhou 310027, China.

^4^ College of Optical Science and Engineering, Zhejiang University, Hangzhou 310027, China

^5^ Key Laboratory of Optoelectronic Chemical Materials and Devices of Ministry of Education, Jianghan University, Wuhan 430056, China.

E-mail: yudazhao@zju.edu.cn


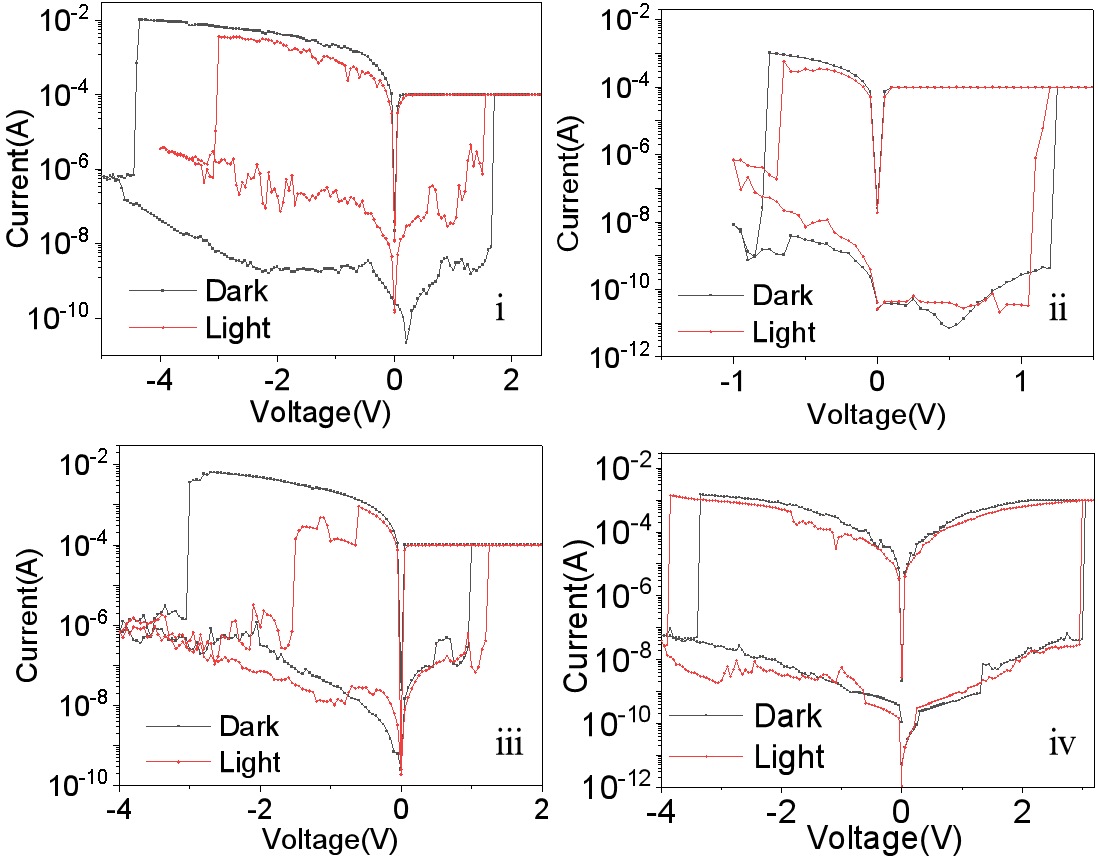


**Figure S1**. Optoelectronic resistance switching of the Au/hBN/Au RRAM illuminated by light with wavelength of 600 nm.


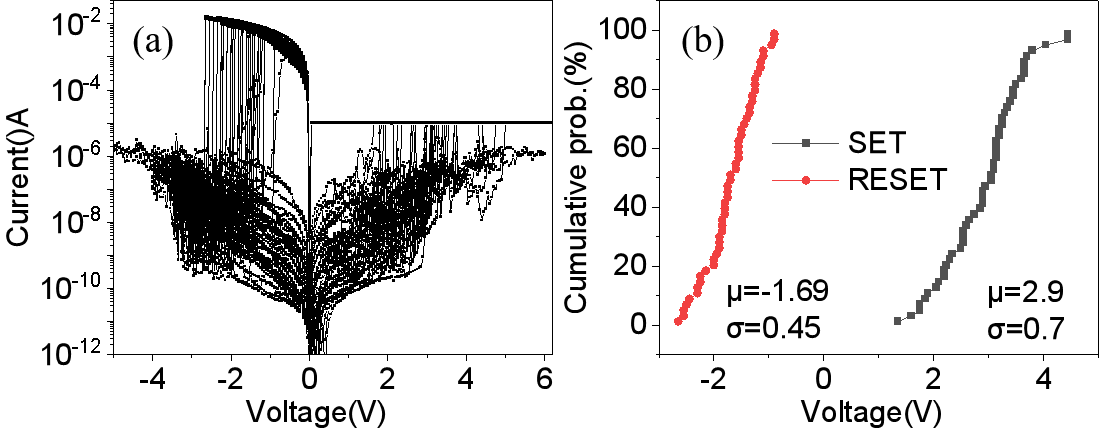


**Figure S2**. (a) 100 direct current *I*-*V* cycles of Au/hBN/Au optoelectronic RRAM and (b) RESET and SET voltage distribution


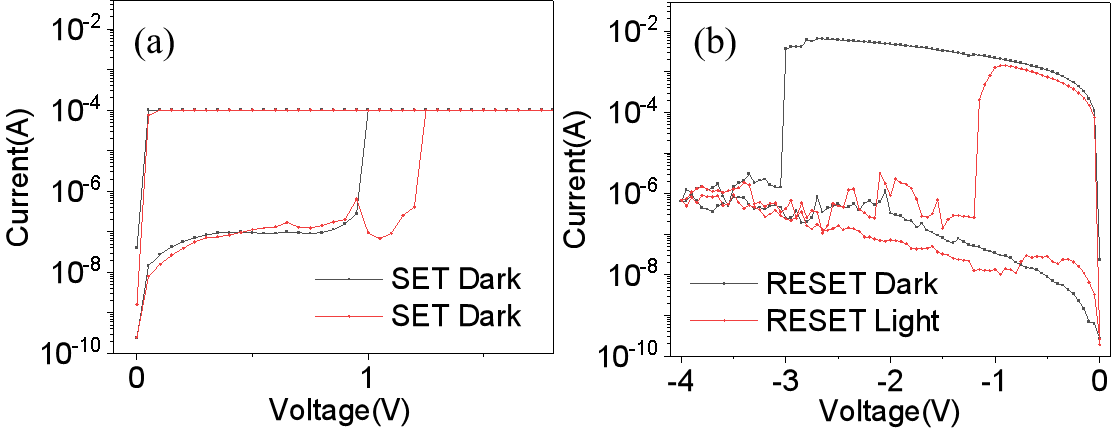


**Figure S3.** (a) SET under dark, and (b) RESET under dark & light with wavelength of 600 nm, respectively.


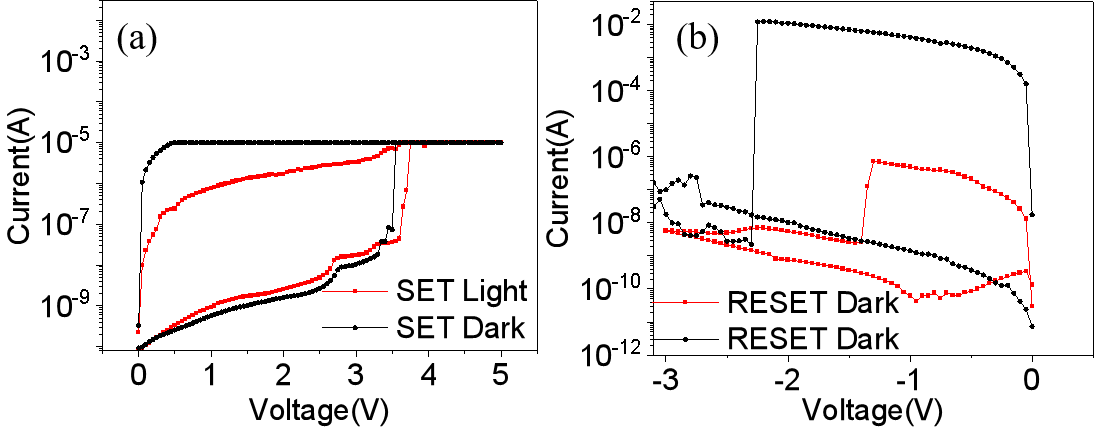


**Figure S4.** (a) SET under dark & light with wavelength of 600 nm, respectively, and (b) RESET under dark.


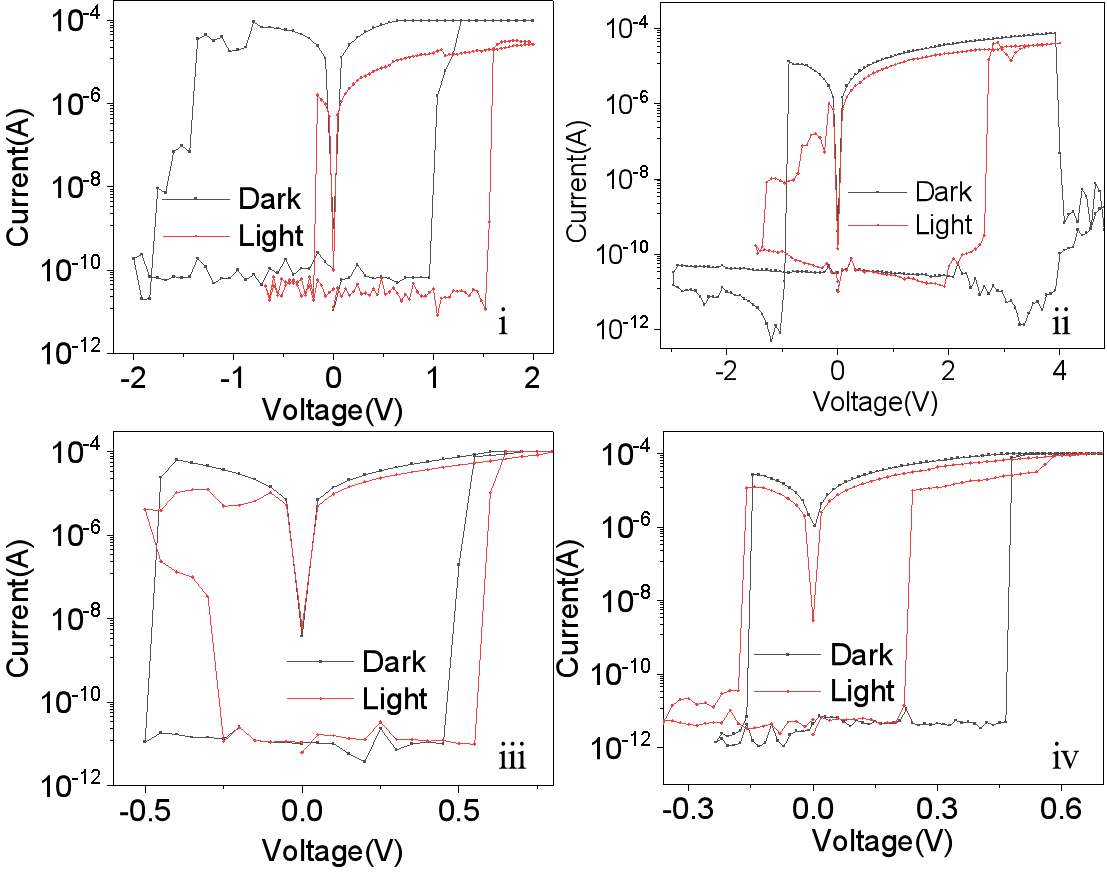


**Figure S5.** Optoelectronic resistance switching of the Ag/hBN/Au RRAM illuminated by light with wavelength of 400 nm.


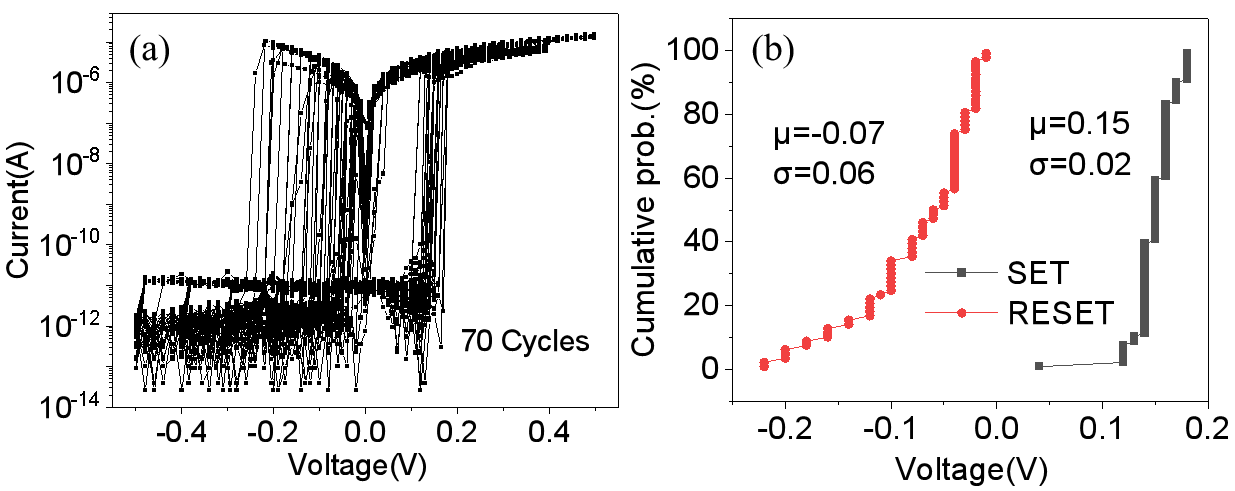


**Figure S6.** (a) 70 direct current *I*-*V* cycles of Ag/hBN/Au optoelectronic RRAM and (b) RESET and SET voltage distribution.


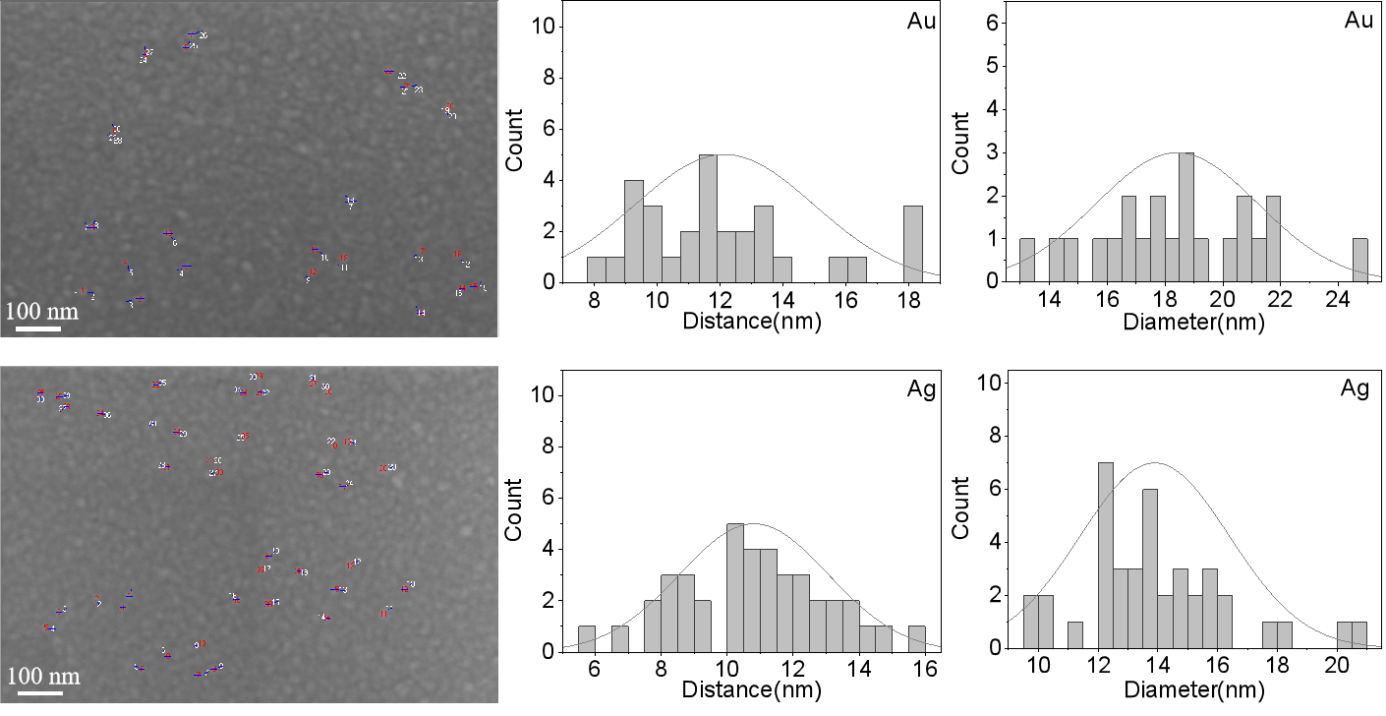


**Figure S7.** The statistical analysis of diameter and distance of Au and Ag nanoparticles in Au and Ag thin film top electrode according to SEM images.


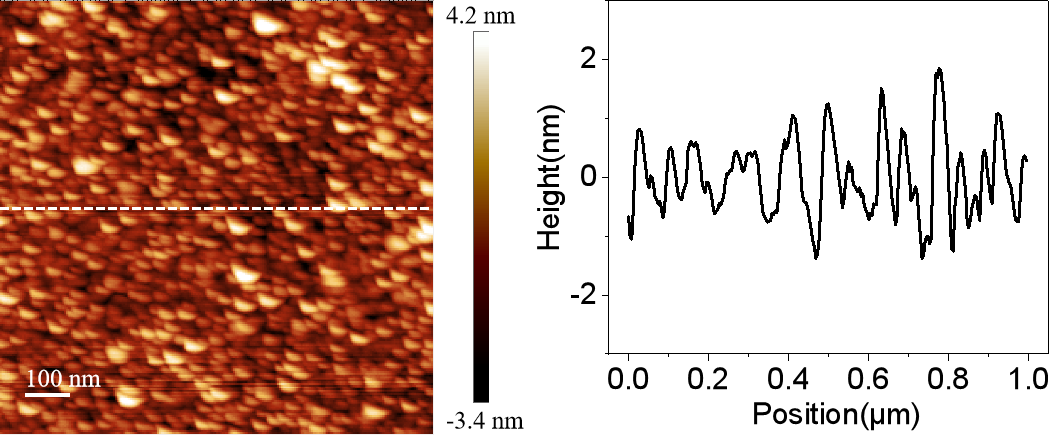


**Figure S8.** AFM images and the roughness (RMS 1.74 nm) of 5 nm thickness top electrode Au film.


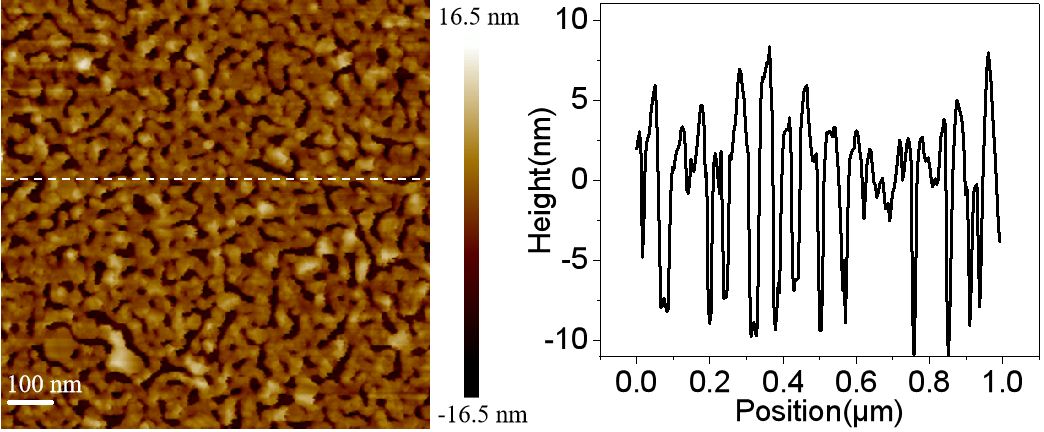


**Figure S9.** AFM images and the roughness (RMS 4.385 nm) of 5 nm thickness top electrode Ag film.


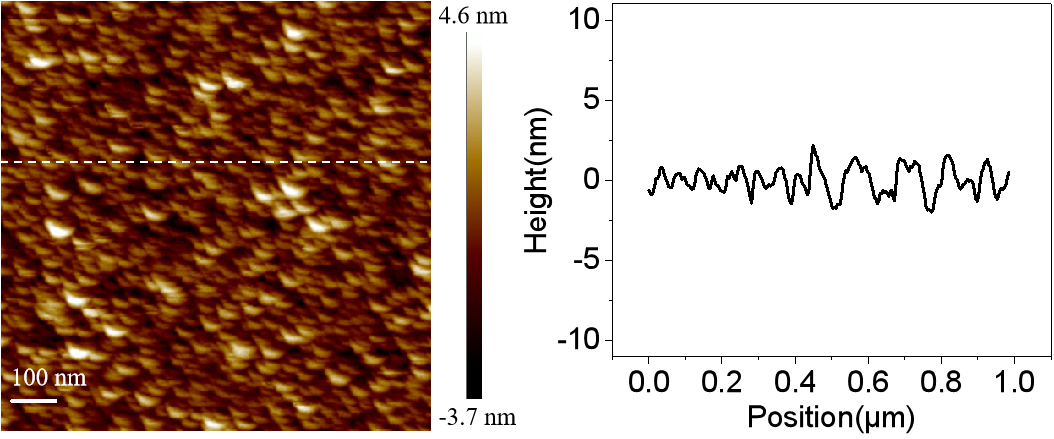


**Figure S10.** AFM images and the roughness (RMS 0.67 nm) of bottom electrode Ti/Au film.


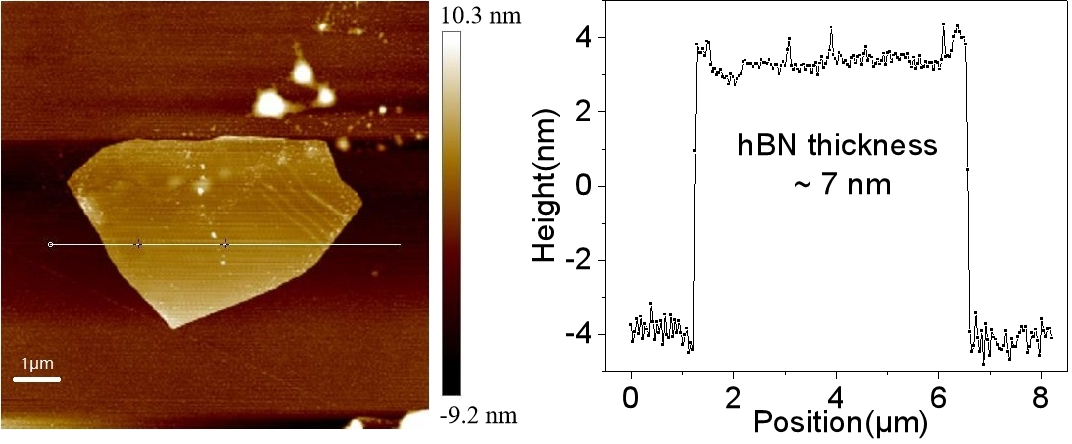


**Figure S11.** AFM images and the thickness (7 nm) of h-BN.


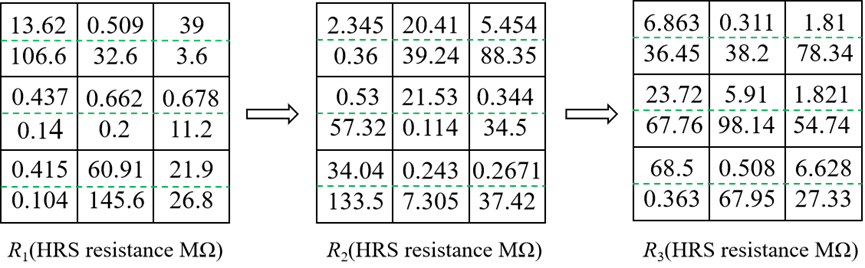


**Figure S12.** The uniqueness calculation of PUF.


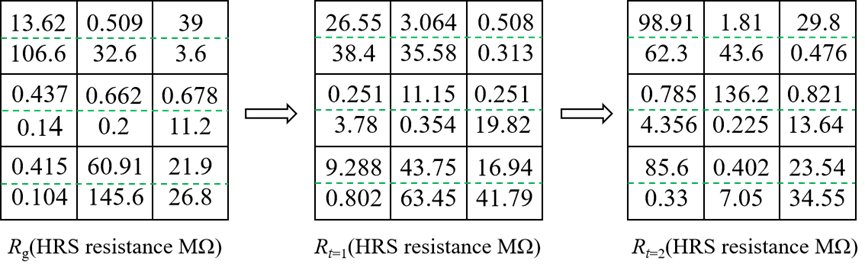


**Figure S13.** The stability calculation of PUF.


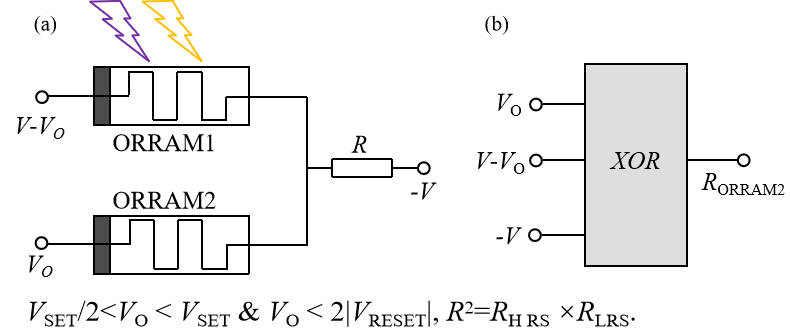


**Figure S14.** (a) equivalent circuit and (b) block diagram of experimental XOR operation.

Two ORRAMs linking in parallel with top bit lines (BLs) are connected to a resistor at the bottom through word lines (WLs). The voltage *V* on ORRAM1 is the input signal *p*. The resistance in HRS and LRS (under light irradiation) of ORRAM1 is the signal *q*. The voltage should comply with the requirements of *V*_SET_/2 < *V*_O_ < *V*_SET_ and *V*_O_ < 2|*V*_RESET_| to avoid mis-operation. The resistor is *R*^2^ = *R*_LRS_ × *R*_HRS_. The set voltage of Au/hBN/Au and Ag/hBN/Au ORRAM are with a mean value of 0.15 V (2.9 V), and a standard deviation of 0.02 V (0.7 V), shown in Figure S2b, S6b. The result p⊕q is accumulated in ORRAM2 resistance of HRS and LRS. The truth table of XOR logic is shown in Table 1.

**Table S1.** The truth table of XOR operation.

| *p* | *q* | *p*⊕*q* |
| --- | --- | --- |
| 0(*V*=0) | 0(ORRAM1=HRS) | 0(ORRAM2=HRS) |
| 1(*V*=*V*_O_) | 0(ORRAM1=HRS) | 1(ORRAM2=LRS) |
| 0(*V*=0) | 1(ORRAM1=LRS Light) | 1(ORRAM2=LRS) |
| 0(*V*=*V*_O_) | 1(ORRAM1=LRS Light) | 0(ORRAM2=HRS) |

**Supplementary Notes**

To quantitatively evaluate the PUF performance, we characterize our RRAM devices with several key parameters, including uniformity, uniqueness, and stability.

(1) Uniformity

The PUF uniformity refers to the probability that 1 and 0 appear in all responses generated by the same PUF entity. In the manuscript, we calculate the Hamming weight of our device, indicating the uniformity of the PUF key with the value of 0.5, which is the ideal value of the Hamming weight of 0.5.

$$Hamming weight=\frac{1}{N}\sum_{i=1}^{N} K^{i} (1)$$

where *N* is the given size of the binary array and *K^i^* refers to the *i*th bit.

(2) Uniqueness

The PUF uniqueness refers to the difference between the responses generated by multiple PUF entities with the same structure but independent of each other. The uniqueness is calculated to be 0.518 according to equation (2) and Figure R1, which is close to the ideal value of 0.5.

$$Uniqueness=\frac{2}{k(k-1)}\sum_{i=1}^{k-1} \sum_{j=i+1}^{k} \frac{HD(R_{i},R_{j})}{n} (2)$$

where *k* indicates that a total of *k* PUF entities participate in the test, *HD*(*R_i_*, *R_j_*) represents the inter-chip Hamming distance between the *i*th and *j*th PUF key, and *n* represents the bit width of the output response of each PUF entity.

(3) Stability (Reliability)

The PUF stability refers to the difference between the PUF output responses when the external factors change when the same PUF entity continuously inputs the same challenge. The uniqueness is calculated to be 0.903 according to equation (3) and Figure R2, which is close to the ideal value of 1.

$$Reliability=1-\frac{1}{m}\sum_{t=1}^{m} \frac{HD(R_{g},R_{t})}{n} (3)$$

where *m* represents the number of measurements, *HD*(*R_g_*, *R_t_*) represents the on-chip Hamming distance, *R_g_* is a reference response measured under certain circumstances, *R_t_* is the response obtained after inputting the same challenge for *t* times, *n* is the bit width of the PUF entity output response.
